# Ultrasonographic Features of Hip Joints in Mucopolysaccharidoses Type I and II

**DOI:** 10.1371/journal.pone.0123792

**Published:** 2015-04-29

**Authors:** Zbigniew Żuber, Agnieszka Jurecka, Agnieszka Różdżyńska-Świątkowska, Agata Migas-Majoch, Agnieszka Lembas, Beata Kieć-Wilk, Anna Tylki-Szymańska

**Affiliations:** 1 Department of Pediatrics, St. Louis Regional Children’s Hospital, Cracow, Poland; 2 Department of Pediatrics, Nutrition and Metabolic Diseases, The Children’s Memorial Health Institute, Warsaw, Poland; 3 Department of Genetics, University of Gdańsk, Gdańsk, Poland; 4 Anthropology Laboratory, The Children’s Memorial Health Institute, Warsaw, Poland; 5 Department of Radiology, The Children’s Memorial Health Institute, Warsaw, Poland; 6 Department of Metabolic Diseases, Medical College Jagiellonian University, Krakow, Poland; Queen Mary University of London, UNITED KINGDOM

## Abstract

**Objectives:**

The primary aim of this study was to assess the ultrasonographic features of hip joints in patients with mucopolysaccharidosis (MPS) type I and II in comparison with healthy population. The secondary aims were to correlate these features with clinical measures and to evaluate the utility of ultrasound in the diagnosis of MPS disease.

**Materials and Methods:**

Sixteen MPS I (n = 3) and II (n = 13) patients were enrolled in the present study and underwent clinical and radiological evaluation, and bilateral high-resolution ultrasonography (US) of hip joints. The distance from the femoral neck to joint capsule (synovial joint space, SJS), joint effusion, synovial hyperthrophy, and local pathological vascularization were evaluated. The results were compared to the healthy population and correlated with clinical and radiological measures.

**Results:**

1. There was a difference in US SJS between children with MPS disease and the normative value for healthy population (7mm). Mean values of SJS were 15.81 ± 4.08 cm (right hip joints) and 15.69 ± 4.19 cm (left joints). 2. No inflammatory joint abnormalities were detected in MPS patients. 3. There was a clear correlation between US SJS and patients’ age and height, while no clear correlation was observed between SJS and disease severity.

**Conclusions:**

1. Patients with MPS I and II present specific features in hip joint ultrasonography. 2. The data suggests that ultrasonography might be effective in the evaluation of hip joint involvement in patients with MPS and might present a valuable tool in facilitating the diagnosis and follow up of the disease.

## Introduction

Mucopolysaccharidoses (MPSs) are a group of lysosomal storage disorders caused by a deficient activity of enzymes responsible for the catabolism of glycosaminoglycans (GAGs) leading to a short stature and severe joint and bone disease [1]. Mucopolysaccharidosis type I (MPS I) is caused by a deficient activity of alpha-L-iduronidase (IDUA; EC 3.2.1.76) and is divided into three subtypes based on the severity of symptoms: Hurler syndrome (severe, OMIM 607016), Hurler–Scheie syndrome (intermediate, OMIM 607015), and Scheie syndrome (attenuated, OMIM 607016) [1–3]. Mucopolysaccharidosis type II (MPS II, Hunter disease, OMIM 309900) is an X-linked recessive disorder caused by a deficiency of iduronate-2-sulfatase (IDS, EC 3.1.6.13). Hunter syndrome affects primarily males while females are non-manifesting carriers of the condition [1].

MPS disorders are characterized by severe skeletal abnormality including growth failure, abnormal bone structure (*dysostosis multiplex*), and severe articular cartilage and joint disease because glycosaminoglycans are fundamental in connective tissue formation, structure and function. The underlying cause of degenerative joint and bone disease is a lack of skeletal remodeling, disordered endochondral and intramembranous ossification, disruption of normal elastogenesis and the infiltration by GAGs of the ligaments, tendons, joint capsules and other tissue structures [4–6]. GAG storage in MPS induces a complex sequence of molecular abnormalities leading to inflammation, apoptosis (cartilage), and hyperplasia (synovial membranes), resulting in poorly organized and metabolically abnormal connective tissue matrices [7–10].

Mucopolysaccharidoses are traditionally evaluated by conventional radiography due to specific changes in the structure and shape of bones. The use of musculoskeletal ultrasound (US) in rheumatology clinical practice allows rheumatologists to diagnose, prognosticate and monitor disease outcome in rheumatoid arthritis [11–13]. It has proven earlier assessment of synovial, cartilage and bone abnormalities than conventional radiology. Numerous studies have also demonstrated that ultrasonographic examination of joints is more sensitive than clinical physical examination [14]. Despite this, there are no studies about the ultrasound investigation of joints in patients with MPS disease.

This is the first ultrasound study of hip joints in mucopolysaccharidoses. The primary aim of this study was to assess the ultrasonographic features of hip joints in patients with MPS type I and II in comparison with healthy population. The secondary aims were to correlate these features with disease severity and to evaluate the utility of ultrasound in the diagnosis of MPS disease.

## Material and Methods

The study objectives were as follows

1. to assess the ultrasonographic features of hip joints in patients with MPS I and II in comparison to healthy population
2. to assess the ultrasonographic features of hip joints in relation to disease severity in patients with MPS I and II
3. to evaluate the utility of ultrasound in the diagnosis of MPS disease

### Study subjects

We performed a prospective and cross-sectional study including 16 male patients (mean age 15.1 years) with a diagnosis of MPS I (n = 3, age 11 and 32 years) or II (n = 13, age range 6–34 years) confirmed by biochemical and molecular analyses (Table 1). All patients were enrolled at the Department of Pediatrics, St. Louis Regional Children’s Hospital, Cracow, Poland.

**Table 1:** Demographic characteristics of 16 patients with mucopolysaccharidoses.

Patient	Disease	Patient’s weight	Patient’s height
(current age)	(phenotype*)	(kg)	(cm)
1 (32)	MPS II (attenuated)	56.3	149.3
2 (10)	MPS II (severe)	40.0	133
3 (14)	MPS II (severe)	38.4	136
4 (8)	MPS II (severe)	32.4	122
5 (12)	MPS II (severe)	26.7	122
6 (7)	MPS II (severe)	24.1	120
7 (6)	MPS II (severe)	26.0	112
8 (31)	MPS II (attenuated)	47.0	151
9 (29)	MPS II (attenuated)	46	150
10 (9)	MPS II (severe)	25.0	123.5
11 (12)	MPS II (severe)	24.0	120.2
12 (6)	MPS II (severe)	25.5	124
13 (9)	MPS II (severe)	23	121
14 (11)	MPS I (Scheie)	30.0	138
15 (34)	MPS I (Scheie)	70.0	171
16 (12)	MPS I (Scheie)	27	143

*Disease classification/severity defined as MPS I—Hurler, Hurler-Scheie, Scheie; MPS II—severe = neuronopathic, attenuated = non-neuronopathic.

### Methods

The US evaluation in all patients was performed bilaterally on hip joints. Ultrasound images were obtained with a Philips model HD 11 XE with a 7.5–12 MHz liner transducer (accuracy 0.1 mm). A standardized procedure similar to that used by other investigators was followed [11,12], a ventral, longitudinal approach was chosen for the hip [11,12].

The following features were assessed: femoral necks, joint cavity, joint capsule (shape, course, thickness, adhesion to femoral neck and head), the distance between femoral neck and joint capsule (the so-called synovial joint space, SJS), echogenicity of joint capsules, synovial fluid (presence or lack of thereof), synovial hyperthrophy, and joint vascularization (using Color Doppler and Power Doppler).

All MPS patients were evaluated on a gray scale of echogenicity and compared to the healthy children.

Both Color Doppler and Power Doppler were used in all patients (PD and CD settings: PRF 0.5–0.9 kHz, gain setting (dynamic range) 20–40 db, frequency 500 Hz, color box (color gain) 18–30 dB) [15].

Ultrasonography in all MPS patients was performed by the same pediatric rheumatologist trained in musculoskeletal US.

### Statistical analysis

The normative value of distance from the femoral neck to joint capsule is 7 mm (as published by the Polish Ultrasonographic Society and Outcome Measures in Rheumatoid Arthritis Clinical Trial (OMERACT) [16–18]). Mean values and standard deviation of SJS of hip joints were calculated for MPS patients. To compare the relationship between the variables such as body weight, height and age ultrasonography values of SJS of hip joints, the linear correlation with Pearson’s product-moment correlation coefficient was performed.

### Ethical Consideration

The protocol was approved by the human-subjects institutional review board at St. Louis Hospital (Ethics Committee, St. Louis Hospital, Cracow, Poland). Written informed consent had to be provided by the parents or legal guardians.

## Results

### Ultrasonographic features of hip joints in patients with MPS I and II in comparison to healthy population (Figs 1–6)

- all patients presented significantly thickened synovial joint space. Mean values of SJS were 15.81 ± 4.08 cm for right hip joints and 15.69 ± 4.19 cm for left joints. Mean values for both joints were greater than the normative value in healthy population
- all patients presented significantly increased echogenicity of joint capsules in comparison to healthy population
- none of the patients presented any signs of synovitis or increased flow through the joint

**Fig 1:**
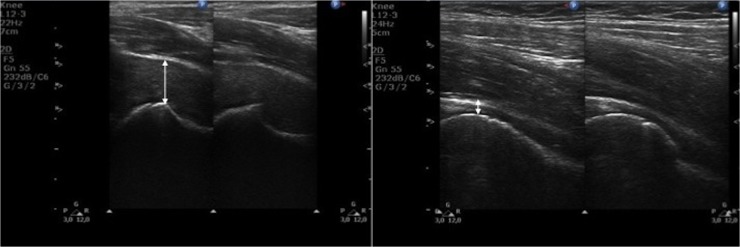
Ultrasound images of hip joints. (Left) Longitudinal scan of hip joint in a 14-year-old patient with a severe phenotype of MPS II. (Right) Longitudinal scan of hip joint in a 14-year-old healthy child. Arrows shows differences in the distance from the femoral neck to joint capsule (synovial joint space, SJS).

**Fig 2:**
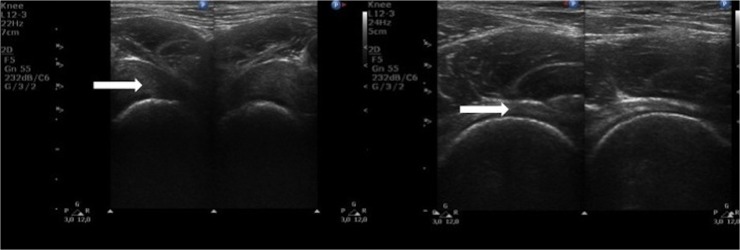
Ultrasound images of hip joints. (Left) Transverse scan of hip joint a 14-year-old patient with a severe phenotype of MPS II. (Right) Longitudinal scan of hip joint in a 14-year-old healthy child. Arrows shows differences in the distance from the femoral neck to joint capsule (synovial joint space, SJS).

**Fig 3:**
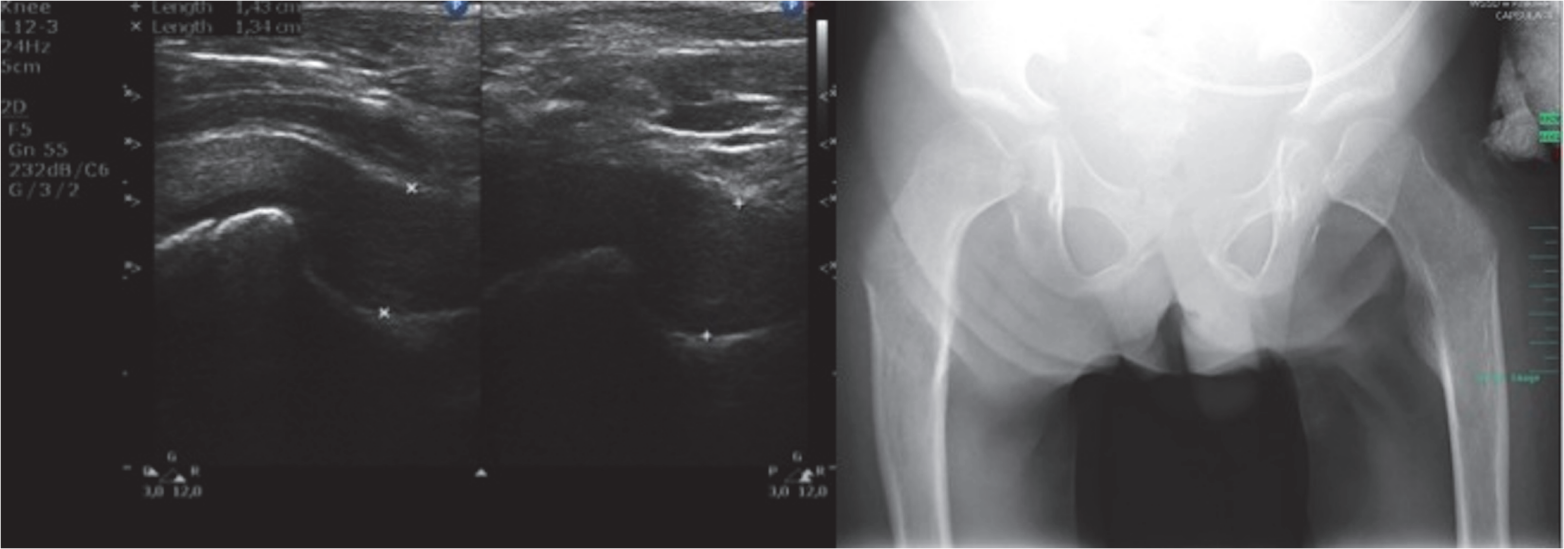
Ultrasound and X-ray images of hip joints. (Left) Transverse scan of hip joint a 12-year-old patient with a severe phenotype of MPS II. (Right) Radiograph of the pelvis of a 12-year-old patient with a severe phenotype of MPS II: dysostosis multiplex (irregular shape of the pelvis, hypoplastic hip acetabulum, dysplastic hips, osteonecrosis of the femoral heads with flattened acetabula, lopsided head of femur bones).

**Fig 4:**
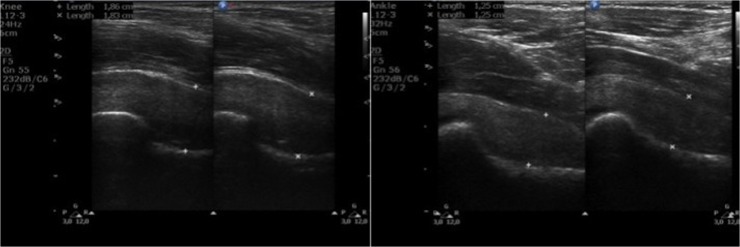
Ultrasound images of hip joints. (Left) Longitudinal scan of hip joint in a 32-year-old patient with an attenuated phenotype of MPS II. (Right) Longitudinal scan of hip joint in a 10-year-old patient with a severe phenotype of MPS II.

**Fig 5:**
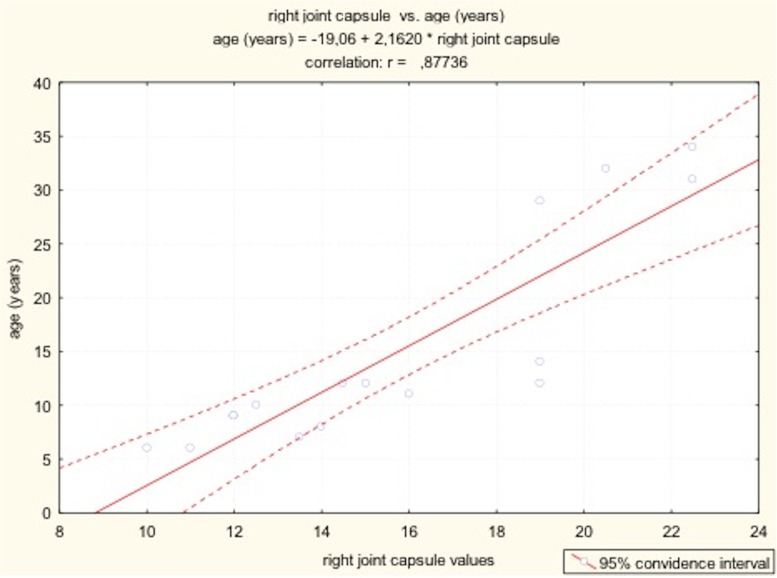
The results of Pearson’s correlation between values of synovial joint space (right joint capsule) and patients’ age and height.

**Fig 6:**
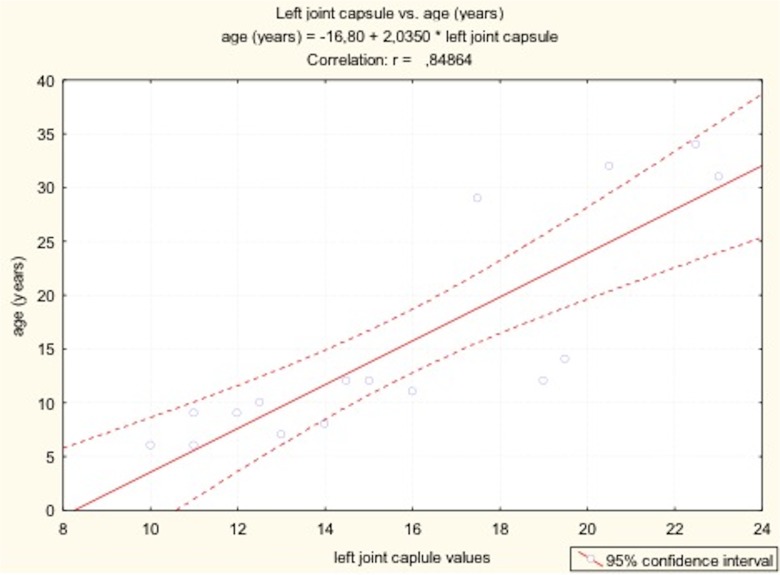
The results of Pearson’s correlation between values of synovial joint space (left joint capsule) and patients’ age and height.

### Ultrasonographic features of hip joints in relation to clinical measures in patients with MPS I and II

- there was a positive correlation between value of SJS and patient’s height and age (Table 2)
- with age, both increase in SJS as well as echogenicity of joint capsule were observed
- no clear correlation was observed between disease severity and value of SJS

**Table 2:** The results of Pearson’s correlation between values of synovial joint space and patients’ age and height.

variable		age (years)	body height
right joint capsule	*Pearson’s r*	0.88	0.66
	*N*	16.00	15.00
	*p value*	**0.00**	**0.01**
left joint capsule	*Pearson’s r*	0.85	0.64
	*N*	16.00	15.00
	*p value*	**0.00**	**0.01**

### Evaluation the utility of ultrasound in the diagnosis of MPS disease

- all patients presented specific ultrasonographic features different than healthy population as well as patients with other rheumatological conditions such as significantly thickened SJS, significantly increased echogenicity of joint capsules, no signs of synovitis or increased flow through the joint

## Discussion

Cartilage is the major area of pathology in mucopolysaccharidoses, leading to poor bone growth, poor joint mobility and painful joints [7,8,10]. Due mainly to lysosomal deposition of GAGs in the chondrocytes, the extracellular matrix of the articular cartilage, the synovia, and the surrounding tissues, MPS patients have stiff joints, contractures and poor mobility [19]. Simonaro et al hypothesized that lysosomal and/or extracellular GAG storage in the MPS disorders induce inflammation and affect the growth of connective tissue cells and other cell types by activating the Toll-like receptor 4 (TLR4) signaling pathway [6]. TLR4 activation in MPS animals resulted in the production of ceramide, a pro-apoptotic lipid and the release of numerous inflammatory cytokines and proteases [9]. Stimulation of MPS connective tissue cells by the inflammatory cytokines causes enhanced secretion of several of the matrix metalloproteinases (MMPs). The imbalance of MMPs over tissue inhibitors of metalloproteinase, the TIMPs, precipitate features of both osteoarthritis as well as rheumatoid arthritis in joints of MPS patients [20].

Mucopolysaccharidoses are traditionally evaluated by conventional radiography. It detects *dysostosis multiplex*, which is a clinical hallmark of almost all different types of MPS with the exception of MPS III [21]. However, some inflammatory and/or primary degenerative joint diseases may mimic the radiologic and clinical findings of MPSs. Also, radiography may be unable to catch early disease signs and depict articular cartilage, especially in attenuated cases. Ultrasound can overcome this barrier since it can visualize articular hyaline cartilage as a well-defined anechoic band lacking internal echoes [22]. Ultrasound can also show pathological signs of articular cartilage in terms of thickness, transparency, and sharpness as well as depict a range of abnormalities from the minimally thickened synovium to severe hyperthrophy with fluid, debris and villi. Owing to better axial and lateral resolution of US, even minute bone surface abnormalities may be depicted. Thus destructive and/or reparative/hypertrophic changes on the bone surface may be seen before they are apparent on plain x rays or even magnetic resonance imaging [23]. Doppler ultrasound additionally allows the visualization of microvascularity within joint cavity and periarticular tissue providing information about the presence or absence of flow through the joint. Musculoskeletal ultrasound has nowadays become an established imaging technique for the diagnosis and follows up of patients with rheumatic diseases. Despite this, so far, there are no studies about the ultrasound investigation of joints in patients with MPS disease.

Our data confirm usefulness of US in imaging MPS changes in hip joints of patients with MPS disease. All patients, regardless of disease progression, presented specific ultrasonographic findings such as significantly thickened synovial joint space with significantly increased echogenicity, and no signs of synovitis or increased flow through the joint. Thickening of the synovial joint space was not dependent on disease severity, but rather length of the disease process. Disease severity is assessed clinically and is associated with mental retardation, while thickening of the SJS is a result of glycosaminoglycan storage in the joints.

Our findings suggest that ultrasonography of hip joints might be effective in the evaluation of hip joint involvement in patients with MPS I and II and might be useful in facilitating the differential diagnosis of MPS disease with other rheumatic diseases, follow up of these diseases and assessment of efficacy of the treatment. Further studies are needed to confirm it. Because bone and joint manifestations and skeletal abnormalities are early and prominent features of MPSs, even in attenuated and mild patients, sooner or later (but fairly often before their underlying illness has been recognized) rheumatologists play a key role in disease recognition and timely diagnosis [24,25]. Consequently, it is important that rheumatologists are aware of the clinical manifestations that could be related to MPS diseases, what else to look for and what diagnostic procedures are available [24]. Ultrasonography has considerable advantages over other imaging methods, including non-invasiveness, speed of performance, relatively low costs, ability to scan multiple joints, repeatability, and high patient acceptability [26]. US of hip joints in older children as well as adults could be included into the diagnostic algorithm of patients with musculoskeletal symptoms.

## Conclusions

1. Patients with MPS I and II present specific features in hip joint ultrasonography.
2. The presence of these features should lead to the suspicion of MPS disease.

## References

[1] Neufeld EF, Muenzer J. The mucopolysaccharidoses In: Valle D, Beaudet AL, Vogelstein B, Kinzler KW, Antonarakis SE et al, editors. The online metabolic and molecular basis of inherited disease. New York: McGraw-Hill.

[2] Roubicek M, Gehler J, Spranger J. (1985) The clinical spectrum of alpha-L-iduronidase deficiency. American Journal of Medical Genetics 20: 471–481. 3922223 10.1002/ajmg.1320200308

[3] Clarke LA, Wraith JE, Beck M, Kolodny EH, Pastores GM, Muenzer J, et al (2009) Long-term efficacy and safety of laronidase in the treatment of mucopolysaccharidosis I. Pediatrics 123: 229–240. 10.1542/peds.2007-3847 19117887

[4] Hinek A, Wilson SE (2000) Impaired elastogenesis in Hurler disease: dermatan sulfate accumulation linked to deficiency in elastin-binding protein and elastic fiber assembly. The American journal of pathology 156: 925–938. 10702409 10.1016/S0002-9440(10)64961-9 PMC1876830

[5] Pastores GM, Meere PA (2005) Musculoskeletal complications associated with lysosomal storage disorders: Gaucher disease and Hurler-Scheie syndrome (mucopolysaccharidosis type I). Current Opinion in Rheumatology 17: 70–78. 15604908 10.1097/01.bor.0000147283.40529.13

[6] Simonaro CM, D'Angelo M, He X, Eliyahu E, Shtraizent N, Haskins ME, et al (2008) Mechanism of glycosaminoglycan-mediated bone and joint disease: implications for the mucopolysaccharidoses and other connective tissue diseases. The American journal of pathology 172: 112–122. 18079441 10.2353/ajpath.2008.070564 PMC2189614

[7] Simonaro CM, D'Angelo M, Haskins ME, Schuchman EH (2005) Joint and bone disease in mucopolysaccharidoses VI and VII: identification of new therapeutic targets and biomarkers using animal models. Pediatric Research 57: 701–707. 15746260 10.1203/01.PDR.0000156510.96253.5A

[8] Simonaro CM, D'Angelo M, He X, Eliyahu E, Shtraizent N, Haskins ME, et al (2008) Mechanism of Glycosaminoglycan-Mediated Bone and Joint DiseaseImplications for the Mucopolysaccharidoses and Other Connective Tissue Diseases. The American Journal of Pathology 172: 112–122. 18079441 10.2353/ajpath.2008.070564 PMC2189614

[9] Simonaro CM, Ge Y, Eliyahu E, He X, Jepsen KJ, Schuchman EH (2010) Involvement of the Toll-like receptor 4 pathway and use of TNF-alpha antagonists for treatment of the mucopolysaccharidoses. Proceedings of the National Academy of Sciences of the United States of America 107: 222–227. 10.1073/pnas.0912937107 20018674 PMC2806747

[10] Simonaro CM, Haskins ME, Schuchman EH (2001) Articular chondrocytes from animals with a dermatan sulfate storage disease undergo a high rate of apoptosis and release nitric oxide and inflammatory cytokines: a possible mechanism underlying degenerative joint disease in the mucopolysaccharidoses. Laboratory investigation; a journal of technical methods and pathology 81: 1319–1328. 11555679 10.1038/labinvest.3780345

[11] Eich G, Halle F, Hodler J, Seger R, Willi U (1994) Juvenile chronic arthritis: imaging of the knees and hips before and after intraarticular steroid injection. Pediatr Radiol 24: 558–563. 7724276 10.1007/BF02012732

[12] Fedrizzi MS, Ronchezel MV, Hilario MO, Lederman HM, Sawaya S, Goldenberg J, et al (1997) Ultrasonography in the early diagnosis of hip joint involvement in juvenile rheumatoid arthritis. Journal of Rheumatology 24: 1820–1825. 9292810

[13] Eberhardt K, Fex E, Johnsson K, Geborek P (1995) Hip involvement in early rheumatoid arthritis. Annals of the Rheumatic Diseases 54: 45–48. 7880121 10.1136/ard.54.1.45 PMC1005511

[14] Dougados M, Jousse-Joulin S, Mistretta F, d'Agostino MA, Backhaus M, Bentin J, et al (2010) Evaluation of several ultrasonography scoring systems for synovitis and comparison to clinical examination: results from a prospective multicentre study of rheumatoid arthritis. Annals of the Rheumatic Diseases 69: 828–833. 10.1136/ard.2009.115493 19740905

[15] Wakefield R, D'Agostino MA (2010) Essential applications of musculoskeletal ultrasound in rheumatology Philadelphia: Saunders Elsevier.

[16] Jakubowski W (2011) Standardy Badań Ultrasonograficznych Polskiego Towarzystwa Ultrasonograficznego: Medbook.

[17] Ciechomska A, Andrysiak R, Serafin-Król M (2005) Ultrasonography, magnetic resonance imaging and conventional radiography of bone erosions in rheumatoid arthritis—a comparative study. Reumatologia 43: 301–309.

[18] Jeka S, Murawska A (2009) Ultrasonografia błony maziowej w chorobach reumatycznych. Reumatologia 47: 339–343.

[19] Oussoren E, Brands MM, Ruijter GJ, der Ploeg AT, Reuser AJ (2011) Bone, joint and tooth development in mucopolysaccharidoses: Relevance to therapeutic options. Biochimica et Biophysica Acta 1812: 1542–1556. 10.1016/j.bbadis.2011.07.013 21827850

[20] Brands MM, Oussoren E, Ruijter GJ, Vollebregt AA, van den Hout HM, Joosten KF, et al (2013) Up to five years experience with 11 mucopolysaccharidosis type VI patients. Molecular Genetics and Metabolism 109: 70–76. 10.1016/j.ymgme.2013.02.013 23523338

[21] Beck M, Muenzer J, Scarpa M (2010) Evaluation of disease severity in mucopolysaccharidoses. J Pediatr Rehabil Med 3: 39–46. 10.3233/PRM-2010-0100 21791828

[22] Naredo E, Acebes C, Moller I, Canillas F, de Agustin JJ, de Miguel E, et al (2009) Ultrasound validity in the measurement of knee cartilage thickness. Annals of the Rheumatic Diseases 68: 1322–1327. 10.1136/ard.2008.090738 18684742

[23] Backhaus M, Kamradt T, Sandrock D, Loreck D, Fritz J, Wolf KJ, et al (1999) Arthritis of the finger joints: a comprehensive approach comparing conventional radiography, scintigraphy, ultrasound, and contrast-enhanced magnetic resonance imaging. Arthritis and Rheumatism 42: 1232–1245. 10366117 10.1002/1529-0131(199906)42:6<1232::AID-ANR21>3.0.CO;2-3

[24] Coppa GV (2011) Why should rheumatologists be aware of the mucopolysaccharidoses? Rheumatology 50. 10.1093/rheumatology/ker391 22210664

[25] Lampe C, Bellettato CM, Karabul N, Scarpa M (2013) Mucopolysaccharidoses and other lysosomal storage diseases. Rheumatic Diseases Clinics of North America 39: 431–455. 10.1016/j.rdc.2013.03.004 23597973

[26] Naredo E, Moller I, Moragues C, de Agustin JJ, Scheel AK, Grassi W, et al (2006) Interobserver reliability in musculoskeletal ultrasonography: results from a "Teach the Teachers" rheumatologist course. Annals of the Rheumatic Diseases 65: 14–19. 15941835 10.1136/ard.2005.037382 PMC1797981

